# Venoarterial extracorporeal membrane oxygenation treatment for acute respiratory distress syndrome and non-occlusive mesenteric ischemia due to *Pasteurella multocida*-related sepsis with purpura fulminans: a case report

**DOI:** 10.1186/s12245-023-00493-1

**Published:** 2023-03-09

**Authors:** Aya Fukuhara, Seiko Fushimi, Masatoshi Nakata, Jumpei Takamatsu

**Affiliations:** Department of Emergency Medicine and Critical Care, Kansai Rosai Hospital, 3-1-69, Inabaso, Amagasaki, Hyogo Japan

**Keywords:** Acute respiratory distress syndrome, Disseminated intravascular coagulation, Septic shock, Acute kidney injury, Purpura fulminans, *Pasteurella multocida*

## Abstract

**Background:**

*Pasteurella multocida*-related sepsis can cause purpura fulminans (PF), a rare thrombotic disorder that often presents acutely and is potentially fatal. As a consequence of disseminated intravascular coagulation, this hematological emergency originates from micro-thrombotic occlusion of peripheral blood vessels and resulting circulatory failure. Thus far, no studies have reported the use of venoarterial extracorporeal membrane oxygenation (VA-ECMO) for saving lives in patients with worsening respiratory and circulatory failure. Moreover, the development of non-occlusive mesenteric ischemia after VA-ECMO has not yet been documented. Here, we describe the case of a 52-year-old female patient with PF and non-occlusive mesenteric ischemia due to *Pasteurella multocida*-related sepsis who received VA-ECMO.

**Case presentation:**

A 52-year-old-female patient presented to the hospital with a week-long fever and worsening cough. Chest radiography findings revealed ground-glass opacity. We made a diagnosis of acute respiratory distress syndrome due to sepsis and initiated ventilatory management. Because respiratory and circulatory parameters were not maintained, VA-ECMO was introduced. After admission, ischemic findings were observed in the periphery of the extremities, and a diagnosis of PF was made. *Pasteurella multocida* was detected in blood cultures. On day 9, the sepsis was cured with antimicrobial treatment. The patient’s respiratory and circulatory status improved, and she was weaned off VA-ECMO. However, on day 16, her stable circulatory system collapsed again, and her abdominal pain worsened. We performed exploratory laparotomy and noted necrosis and perforation of the small intestine. As a result, partial resection of the small intestine was performed.

**Conclusion:**

In this case, VA-ECMO was used to maintain circulatory dynamics during septic shock in a patient with *Pasteurella multocida* infection who developed PF. Surgery was also performed for complicated ischemic necrosis of the intestinal tract, helping save the patient's life. This development illustrated the importance of paying attention to intestinal ischemia during intensive care.

## Background

*Pasteurella* infection in humans occurs as localized infections of the skin and soft tissues after animal bites and scratches. Although rare, sepsis caused by *Pasteurella multocida* can result in purpura fulminans (PF) [1, 2]. The mechanism underlying *Pasteurella* infection is ischemic necrosis of the muscles and skin of the extremities due to micro-thrombotic occlusion of peripheral blood vessels and circulatory failure caused by disseminated intravascular coagulation (DIC) [1, 2].

To date, there have been no reports on the use of venoarterial extracorporeal membrane oxygenation (VA-ECMO) to save the lives of patients with worsening respiratory and circulatory failure due to *Pasteurella* infection-related sepsis. Furthermore, there are no reports of patients who developed non-occlusive mesenteric ischemia (NOMI) and had to undergo emergency surgery after being saved with VA-ECMO.

In this study, we describe the case of a 52-year-old-female patient who had PF and NOMI caused by *Pasteurella multocida*-related sepsis and was treated with VA-ECMO.

## Case presentation

A 52-year-old-female patient presented with a fever and cough for a week that had worsened over 1 week; however, her symptoms worsened, and she was brought to the emergency department. She had no remarkable medical history, medication use, or allergies. Her height, weight, and body mass index were 152 cm, 71.9 kg, and 31.1 kg/m^2^, respectively. Her respiratory rate was 24 breaths/min, oxygen saturation was 92% (oxygen mask with reservoir 15 L/min), blood pressure (BP) was 110/70 mmHg, heart rate (HR) was 118 beats/min, and body temperature was 39.5 °C. Her level of consciousness, using the Glasgow Coma Scale, was E3 (best eye response: eye opening to sound), V5 (best verbal response: oriented), M6 (best motor response: obeys commands).

Findings of arterial blood gas analysis (oxygen mask with reservoir: 15 L/min) were as follows: pH, 7.411; PaCO_2_, 29.3 mmHg; PaO_2_, 63.0 mmHg; HCO_3_^−^, 20.4 mmol/L; base excess, − 5.5 mmol/L; and lactate, 8.1 mmol/L.

**Fig. 1:**
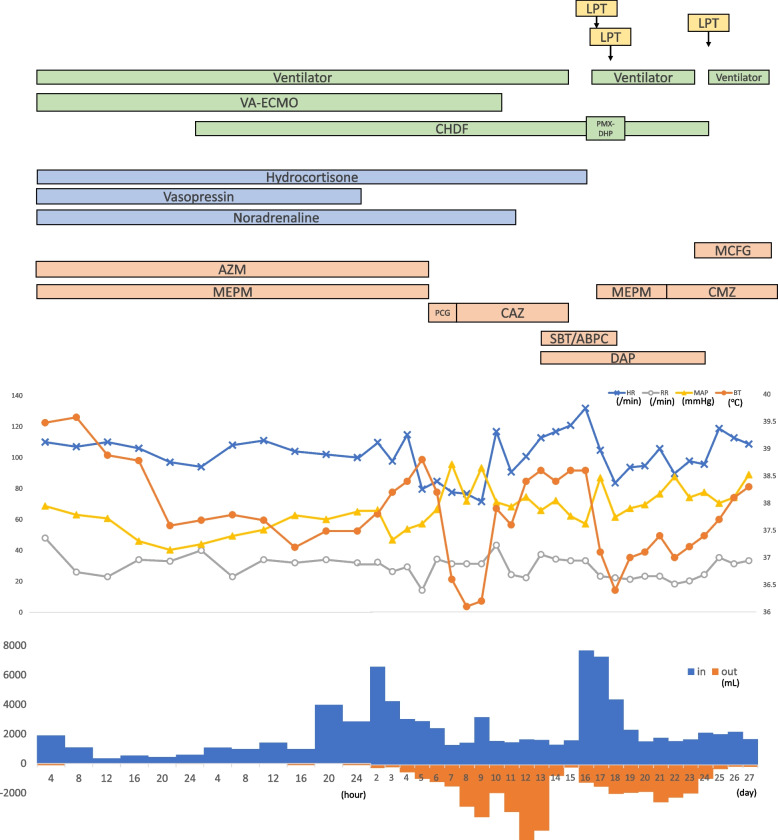
The patient’s clinical course from transportation to third laparotomy. The time series is shown in hours only for day 1 and in days from day 2 onward. VA-ECMO: venoarterial extracorporeal membrane oxygenation; CHDF: continuous hemodiafiltration; PMX-DHP: polymyxin B immobilized fiber column direct hemoperfusion; HR: heart rate; RR: respiratory rate; MAP: mean arterial pressure; BT: body temperature; LPT: laparotomy; MEPM: meropenem; AZM: azithromycin; PCG: penicillin G; CAZ: ceftazidime; SBT/ABPC: sulbactam/ampicillin; DAP: daptomycin; CMZ: cefmetazole; MCFG: micafungin

**Fig. 2:**
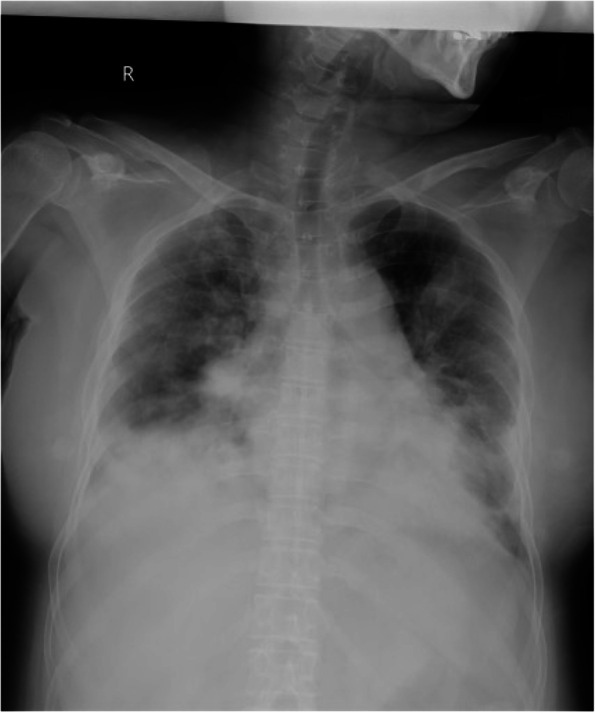
Chest radiography findings show infiltrative shadows on both lung fields

**Fig. 3:**
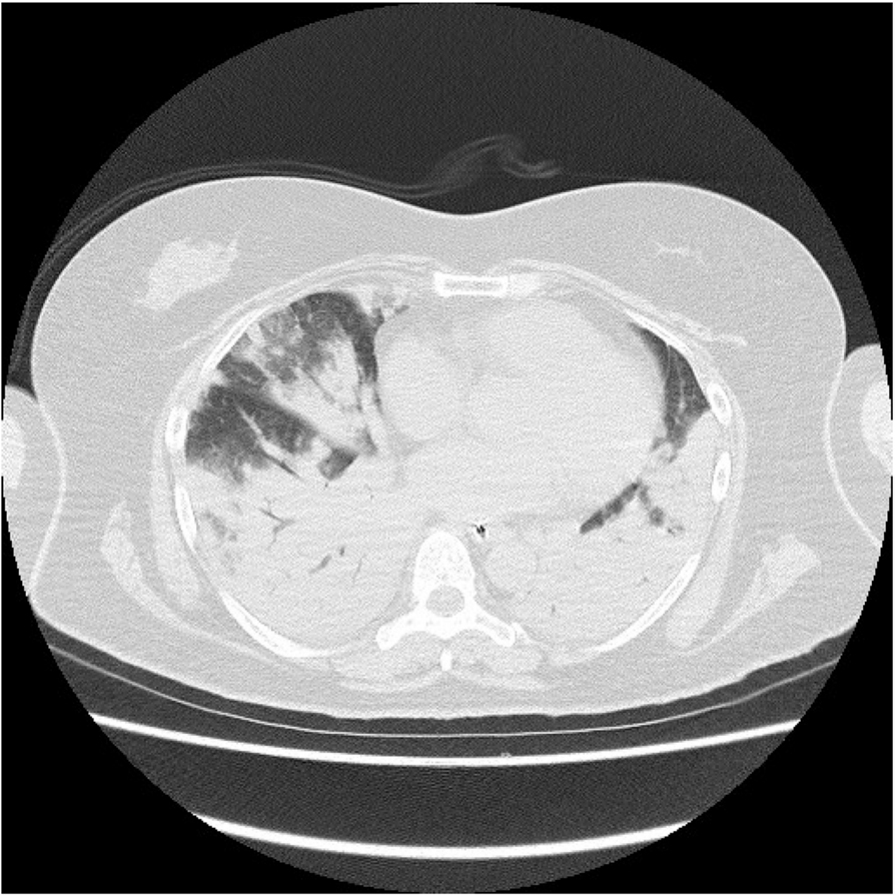
Chest computed tomography findings show infiltrating shadows that predominate in the bilateral lower lung fields

**Fig. 4:**
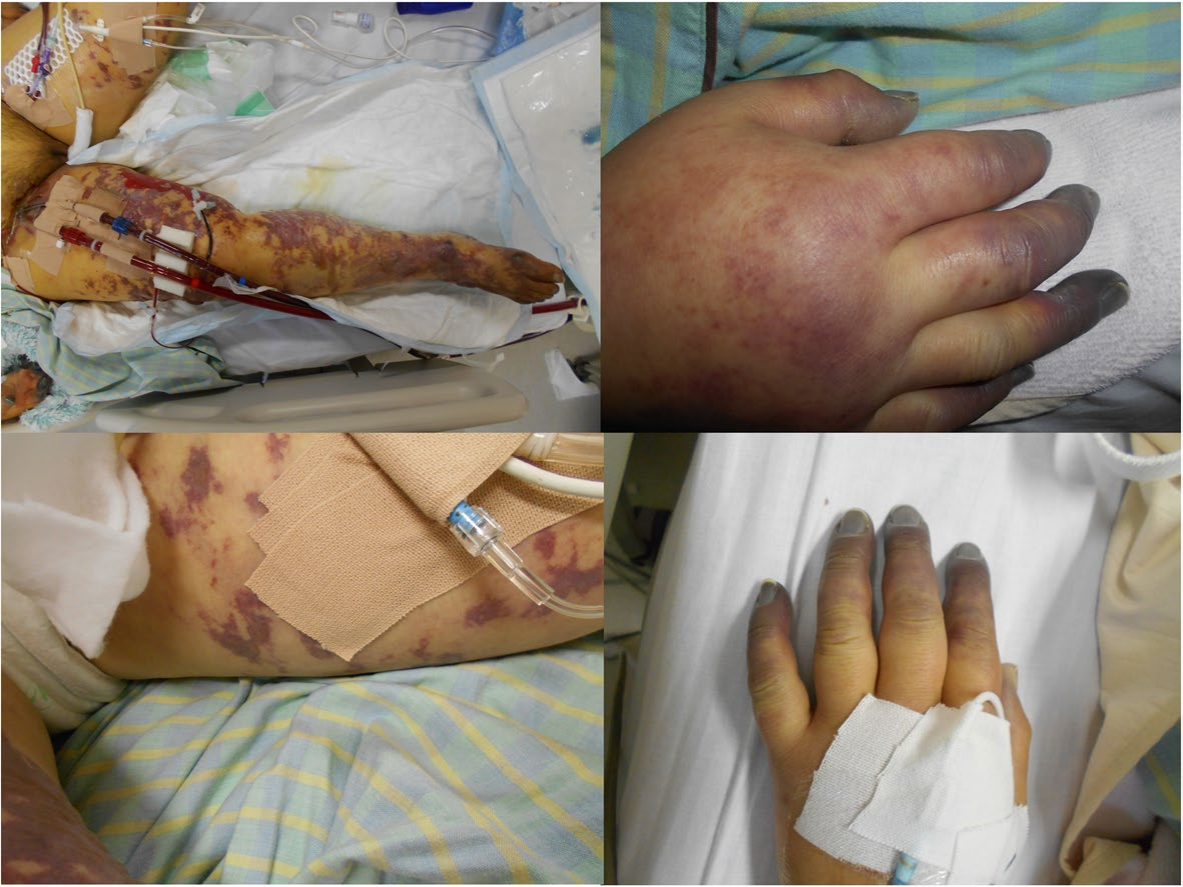
Image of the peripheral extremities shows degeneration due to ischemia, with black coloration. Purpura can be seen on the extremities, especially on the right lower extremity, with commensal extension to the thigh

**Fig. 5:**
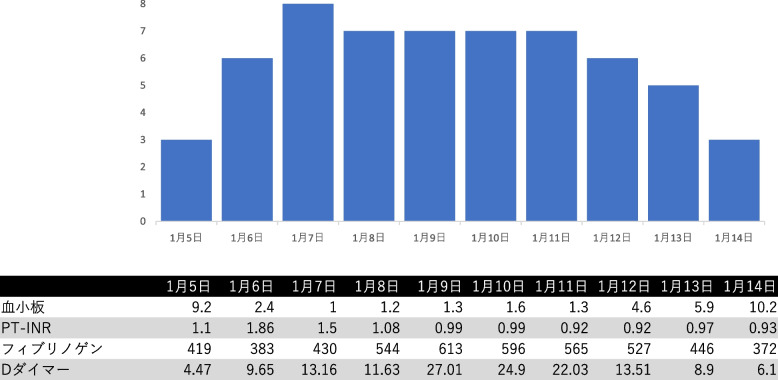
Acute disseminated intravascular coagulation score. The score worsened day by day, peaking on day 3

**Fig. 6:**
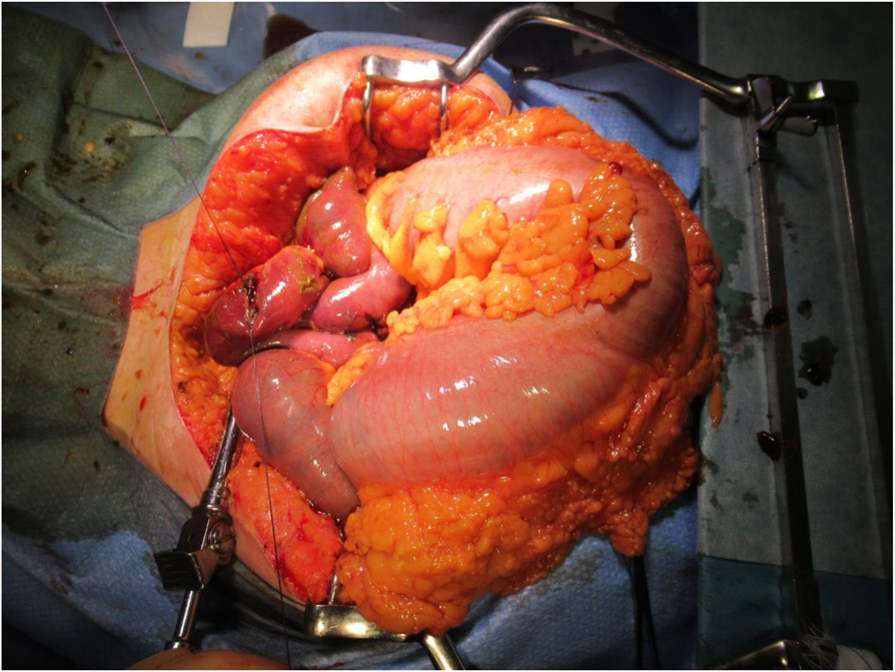
Intraoperative findings. The small intestines show ischemic changes and partial perforation

The clinical course of the patient is shown in Fig. 1. Chest radiography (Fig. 2) findings showed diffuse ground-glass opacities in the bilateral lung fields, and chest computed tomography (CT) (Fig. 3) showed infiltrating shadows in the lower lung fields. Blood test results are presented in Table 1.. An echocardiogram was performed, and an ejection fraction of 65% and wall motion within normal limits helped rule out heart failure. The patient was diagnosed with acute respiratory distress syndrome (ARDS) due to sepsis because of significant inflammatory findings, including a white blood cell count of 1200/µL and a C-reactive protein level of 17.1 mg/dL. The patient was intubated, and ventilatory management was initiated due to poor oxygenation. The Murray score at this time was 4. The patient’s circulatory status was still under the shock state, with a BP of 66/50 mmHg even with catecholamine (noradrenaline 0.013 μg/kg/min) administration. As both respiratory and circulatory status could not be maintained despite ventilatory management, fluid administration, and catecholamine administration, VA-ECMO was started as an adjuvant circulatory therapy. As the patient had anuria, she was considered to have acute kidney injury and was therefore started on continuous hemodiafiltration. Peripheral circulation was poor from the time she was transported from her house to the hospital, and purpura was present in the peripheral extremities (Fig. 4). After the insertion of an arterial line into the right femoral artery and initiation of VA-ECMO, the mottled purpura of the right lower limb worsened. Further deterioration was prevented by inserting a sheath into the right femoral artery toward the periphery to maintain blood flow and peripheral circulation in the right lower limb. The acute DIC score was 4 (platelet count, 92,000/µL; PT-INR prothrombin time-international normalized ratio, 1.10; D-dimer, 4.47); hence, the patient was considered to have complicated DIC (Fig. 5). Because of her circulatory instability, we decided to administer meropenem (MEPM) and azithromycin as broad-spectrum antimicrobials. On day 4, penicillin G (PCG) was added due to the detection of *Pasteurella multocida* in blood cultures. On day 6, PCG was changed to ceftazidime because of proven susceptibility. On day 9, VA-ECMO was withdrawn because the patient’s circulatory status was stable (HR 70 beats/min, BP 120/60 mmHg), remaining so despite reduced support by VA-ECMO. On day 11, enteral feeding was initiated. On day 14, the patient was extubated as the respiratory condition had stabilized. On day 15, the patient presented with abdominal pain. However, there were no obvious findings on contrast-enhanced CT. On day 16, septic shock redeveloped; elevated lactate level led to a suspected diagnosis of intestinal ischemia and, thereafter, a confirmed diagnosis of NOMI. Therefore, an experimental laparotomy was performed. The small intestine was perforated due to ischemia. Partial small bowel resection and open abdominal management were performed for small bowel necrosis and perforation, respectively (Fig. 6). After the operation, Polymyxin B-immobilized fiber column direct hemoperfusion was performed to improve circulatory dynamics. On day 17, no new abnormal findings were found in the second-look surgery; therefore, the separated small intestine was anastomosed, and the abdomen was closed. Subsequently, MEPM was changed to cefmetazole owing to suspected drug-induced leukocytopenia. However, micafungin was additionally administered due to lack of improvement in the inflammatory findings. On day 24, there was no evidence of circulatory disturbance, but a brown discharge from the wound was observed. Therefore, a third emergency laparotomy was performed, which revealed anastomotic leakage. A small-bowel anastomosis resection was subsequently performed. On day 30, the patient’s respiratory status deteriorated again, and she was considered to be having a flare-up. Intra-abdominal infection was suspected as the cause. On day 35, a fourth laparotomy was performed. Owing to anastomotic re-leakage, partial resection of the small intestine and double-barreled stoma were performed. On day 55, the patient underwent amputations of 2–5 fingers of both hands with ischemic necrosis and segmental skin grafting. On day 68, debridement of the right whole metatarsal bones was performed because of abscess formation in the right plantar region. On day 73, owing to delayed wound healing, we decided to amputate the right lower limb. Upon incision, however, the right lower leg revealed itself to be so extensively necrotic that a right femoral amputation was performed. On day 98, MEPM was initiated because of fever and elevated inflammatory findings, suspected to be due to bacteremia. On day 99, incisional abscess drainage was performed because of the presence of a right rectus femoris abscess. The patient continued to have liver dysfunction and gastrointestinal malabsorption, and although it took time for her to stabilize, she underwent stoma closure on day 382 and was discharged home on day 443.

**Table 1.** Laboratory findings at the time of admission

Complete blood count	Reference	
White blood cells	1,200	4000-9000 /μL
Red blood cells	438 × 10^4 ^	380-540× 10^4 ^/μL
Hemoglobin	13.6	11.5-15 g/dL
Platelet	92 × 10^3^	150-350× 10^3^/μL
Coagulation status		
Activated partial thromboplastin time	41.4	26-36 seconds
Prothrombin time-international normalized ratio	1.1	0.8-1.1
Fibrinogen	419	200-400 mg/dL
D-dimer	4.47	0-0.49 μg/mL
Arterial blood gas		
FiO_2_	1.0	
pH	7.394	7.35-7.45
PaCO_2_	32.9	35-45 mmHg
PaO_2_	73.9	80-100mmHg(room air)
HCO_3_^−^	21.1	22-26 mmol/L
Base excess	-4.4	-2 to +2
Lactate	7.4	<2mmol/L
Biochemistry		
Total bilirubin	0.7	0.2-1.2 mg/dL
Aspartate transaminase	139	12-35 U/L
Alanine transaminase	85	5-30 U/L
Alkaline phosphatase	235	109-344 U/L
Lactate dehydrogenase	712	110-240 U/L
Cholinesterase	300	217-491 U/L
Creatine kinase	2007	13-187 U/L
Blood urea nitrogen	13.7	8-20 mg/dL
Creatinine	2.13	0.4-0.8 mg/dL
Sodium	138	mEq/L
Potassium	3.0	3.5-5 mmol/L
Chloride	96	98-110 mmol/L
Total protein	6.3	6.1-8.1 g/dL
Albumin	2.9	3.2-5 g/dL
C-reactive protein	17.1	0-0.3 mg/dL
Blood sugar	121	<140 mg/dL
Hemoglobin A1c	6.5	4.6-6.2 %
Procalcitonin	>100	0-0.49 ng/mL
N-terminal pro-brain natriuretic peptide	2171	0-125 pg/mL

## Discussion and conclusions

*Pasteurella multocida i*s believed to be transmitted to humans through bites and scratches from dogs and cats [1, 2]. In the present case, the dog was kept as a pet; however, the details are unknown. Bacteremia caused by *Pasteurella multocida* predominantly affects the liver and can lead to refractory sepsis and death [1]. After blood infection initiates, sepsis develops in 25–50% of cases, and 30-day mortality is reported to occur in 31.1% of patients [3]. Risk factors for *Pasteurella multocida* infection include diabetes, age over 65 years, immunocompromised status, cirrhosis, and history of chronic lung disease [1, 4]. In the present case, no risk factors were observed, and *Pasteurella multocida* was not considered in the differential diagnosis as the causative organism until culture results were available.

PF is a rapidly evolving syndrome characterized by microvascular thrombosis and hemorrhagic necrosis. The skin findings of PF include characteristic appearance and evolution. PF begins with erythema, which develops as blue-black hemorrhagic necrosis in irregular central areas. It is a term used to describe any clinical presentation of disseminated purpura in patients with the following etiologies [5]. The three most common etiological categories of PF are idiopathic, hereditary, or acquired coagulation abnormalities and sepsis, with sepsis being the most frequent [2]. In this case, the diagnosis of PF associated with sepsis due to *Pasteurella multocida* infection was made based on the clinical history. This history showed a rash characteristic of sepsis patients with PF and the presence of DIC.

PF due to *Pasteurella multocida* infection is rare, with only two cases reported in the literature [1, 2]. Furthermore, we could not find any reports of ARDS due to *Pasteurella multocida* infection that involved successful resuscitation with VA-ECMO; in our case, the patient was weaned off VA-ECMO but subsequently experienced NOMI during the clinical course. Moreover, the patient developed septic shock upon admission, and the possibility of NOMI had to be assumed. This raises the question of whether *Pasteurella* infection causes ARDS. It has been reported that infection in humans can migrate from open wounds and cause various infections of the soft tissues, lungs, joints, and bones [3], but there are no such reports for ARDS. The patient had infiltrative shadows with ground-glass opacity spreading to both lungs from the time she was brought to the hospital, and her respiratory condition was extremely poor. Suction sputum culture detected normal flora. We suspected ARDS rather than pneumonia from the X-ray and CT findings.

It has been said that sepsis is a major cause of ARDS [6]. When the infection is very dramatic, as it was in this case, we tend to think it may be consistent with ARDS in terms of mechanism.

The patient's respiratory condition was critical from the time of admission, and supportive care for breathing was necessary.

Although we were unable to examine in detail the course of death in patients with septic shock due to *Pasteurella multocida*, there was a case reported in which the patient deteriorated over time and died within 24 h [4]. In the other reports that further showed PF, the inability to maintain circulation within a few hours was fatal [1], and even if not fatal, limb amputation awaited the patient [2]. These suggest that there are some fulminant cases. Therefore, supportive care is important to maintain circulatory control until antimicrobial agents are effective.

Since *Pasteurella multocida*-related sepsis is associated with a high mortality rate, it is essential to initiate treatment as early as possible. Even the most severely ill patients, such as the patient in this case who was in a critical condition upon arrival at the hospital and who had PF shortly after admission, have a chance of survival if respiratory and circulatory status can be maintained using VA-ECMO.

In conclusion, VA-ECMO can be used to maintain circulatory status and reduce mortality in patients with PF. However, the presence of intestinal ischemia should always be considered.

## Data Availability

The datasets generated during and/or analyzed during the current study are available from the corresponding author on reasonable request.

## Abbreviations

VA-ECMO: Venoarterial extracorporeal membrane oxygenation
PF: Purpura fulminans
DIC: Disseminated intravascular coagulation
NOMI: Non-occlusive mesenteric ischemia
HR: Heart rate
BP: Blood pressure
CT: Computed tomography
ARDS: Acute respiratory distress syndrome
MEPM: Meropenem
PCG: Penicillin G
